# The associations between internet use time and school performance among Korean adolescents differ according to the purpose of internet use

**DOI:** 10.1371/journal.pone.0174878

**Published:** 2017-04-03

**Authors:** So Young Kim, Min-Su Kim, Bumjung Park, Jin-Hwan Kim, Hyo Geun Choi

**Affiliations:** 1 Department of Otorhinolaryngology-Head & Neck Surgery, CHA Bundang Medical Center, CHA University, Seongnam, Korea; 2 Department of Otorhinolaryngology-Head & Neck Surgery, Korea University Ansan Hospital, Ansan, Korea; 3 Department of Otorhinolaryngology-Head & Neck Surgery, Hallym University College of Medicine, Anyang, Korea; 4 Department of Otorhinolaryngology-Head & Neck Surgery, Hallym University College of Medicine, Seoul, Korea; Universita degli Studi di Perugia, ITALY

## Abstract

Although overuse of the internet has been suggested to be related to poor academic performance, the effects of internet use for education on academic performance showed conflict results in previous studies. Accordingly, the associations of school performance with internet use for study and for general purpose were explored in a large population of Korean adolescents. Cross-sectional data from the 2013 Korean Youth Risk Behaviour Web-based Survey (KYRBWS) were retrieved for 59,105 12- to 18-year-old adolescents. The associations between school performance and internet use were analysed using multinomial logistic regression with complex sampling. Days of physical activity, sex, obesity, region of residence, income level, parental education level, stress, sleep time, smoking, alcohol consumption, drug use, and total study time were recorded and adjusted for as confounders. Higher school performance was positively associated with longer internet use for study (adjusted odds ratio, AOR, of 2+ h [95% confidence interval] = 2.43 [2.10–2.82], 2.02 [1.78–2.30], 1.66 [1.46–1.89], and 1.30 [1.15–1.47] for performance groups A, B, C, and D, respectively, P < 0.001) but negatively associated with longer internet use for general purpose (AOR of 3+ h [95% confidence interval] = 0.68 [0.60–0.78], 0.85 [0.76–0.94], 0.83 [0.75–0.92], and 0.98 [0.89–1.08] for performance groups A, B, C, and D, respectively, P < 0.001). Higher school performance significantly positively correlated with internet use for study but negatively correlated with internet use for general purpose. Academic use of the internet could be a means of achieving good school performance.

## Introduction

Academic performance in adolescence, which subsequently determines individuals’ final educational level, is a crucial factor that has substantial effects on adolescents’ later life. School performance could be represented by academic achievement of school grades and cognitive performance. It is determined by environmental factors such as socioeconomic levels as well as each individual characteristics including aptitude and emotional factors [1,2]. Socioeconomic inequalities, such as economic hardship, difficulty with employment, and activity limitation, have been suggested to be significantly related to education levels [3]. In turn, the socioeconomic differences caused by income level and employment status may result in inequalities in educational levels [4]. Therefore, the socioeconomic gap could widen due to the effects of academic performance in adolescence on later life.

The internet is a versatile means of engaging in social communication, retrieving large amounts of information, and performing leisure activities. Adequately controlled use of the internet as information and communication technologies could provide many opportunities including improvement of academic achievements [5–7]. Internet networks allow us to interact with a wide range of people worldwide. In addition, internet-based learning programs, medical education systems, and health interventions such as cognitive therapy have become widespread, generating positive outcomes [8,9]. Approximately 93% of adolescents in the U.S. have been reported to use the internet and approximately 70% of adolescents in Europe surf online for 2–4 hours per day [10,11]. It was estimated that about 99.9% of Korean adolescents use internet in daily life [12].

The overuse of internet, on the other hands, is uprising concern. Approximately 13.1% of adolescents were categorized as problematic internet user in the 2010 Korean Youth Risk Behavior Web-based Survey (KYRBWBS) [13]. and approximately 21.8% of Korean adolescents have been found to overuse computers for gaming (> 3 hours per day) [14]. Internet overuse may lead to several psychological problems, including depression, attention deficit hyperactivity disorder (ADHD), daytime sleepiness, and self-injurious behaviour [15–20]; additionally, overuse of the internet has been related to detrimental physical health conditions, such as obesity [21,22]. Problematic internet use has been suggested to be related to lower school grades [22]. In an earlier prospective study, internet overuse and school burnout were shown to reciprocally affect each other [23]. In addition to the distraction from schoolwork and the relative shortage of time for academic activities caused by internet overuse, socioeconomic factors such as income, parental education level, and region of residence; psychological factors including stress; and physical factors such as medication use, smoking and alcohol use may mediate the effects of internet use on school performance. These potential confounding factors should therefore be considered when exploring the associations between internet use and school performance. Therefore, there have been attempts to access the problematic internet use considering social and emotional factors in adolescents [24].

Although several previous studies have found that internet use has adverse effects on academic performance, they did not fully consider the purposes of internet use in their analyses [14,25]. Some researchers discriminated the purpose of internet use and investigated their influences on school performance, which showed inconsistent results [26–28]. In addition, many studies focused on extreme cases of internet use, identifying adverse psychological and behavioural outcomes due to problematic internet use [19,20,29]. However, internet provides easily accessible diverse contents, it was predicted that appropriate use of internet, in aspects of its purpose as well as using time could assist education in adolescents. To prove this hypothesis, the relationship between academic performance and internet use according to its purpose and a wide range of use durations were investigated in the Korean adolescent population.

## Materials and methods

### Study population and data collection

The Institutional Review Board of the Korean Centers for Disease Control and Prevention (KCDC) approved this study (2014-06EXP-02-P-A). Written informed consent was obtained from each participant prior to the survey. Because this web-based survey was performed at a school with many participants, the need to obtain informed consent from students’ parents was waived. This consent procedure was approved by the IRB of the KCDC.

This was a cross-sectional nation-wide study using data from the 2013 KYRBWS collected by the KCDC, which were analysed using statistical methods based on designed sampling and adjusted weighting. Korean adolescents in 7^th^ through 12^th^ grade voluntarily and anonymously completed the self-administered questionnaire. The surveys obtained data from South Korean adolescents using stratified, two-stage (schools and classes) clustered sampling based on data from the Education Ministry. Sampling was weighted by statisticians, who calculated the weights post-stratification and considered non-response rates and extreme values.

Out of a total of 72,435 participants, we excluded the following from this study: participants who did not provide information on sleep time or who slept less than 2 hours (9,128 participants); participants who did not record their height or weight (1,597 participants); and participants who did not provide the study time (2,605 participants). Finally, 59,105 participants (29,489 male and 29,616 female) aged 12 through 18 years were included in this study.

### Survey

The understanding, reliability and validity of each question were investigated by the KCDC to verify the applicability of the surveys [30]. The validity and reliability of the KYRBWS have been documented by other studies [31,32].

#### Possible confounders of school performance

The time that adolescents fell asleep and woke up was measured in hours and 10-minute intervals. Duration of sleep was calculated by subtracting the time that participants fell asleep from the time that they woke up. Sleep time was classified into 4 groups: < 5.5 hours; ≥ 5.5 and < 6.5 hours; ≥ 6.5 and < 7.5 hours; and ≥ 7.5 hours. These values were selected because they were identified as the quartile points for sleep time in this study. Days of physical activity were expressed as the number of days in the past 7 days that the adolescents had exercised for more than 60 minutes at an intensity sufficient to increase their heart rate or respiration. Obesity was categorized into 4 groups according to the CDC guidelines for body mass index (BMI, kg/m^2^) for children and teens [33]: obese ≥ 95^th^ percentile; overweight ≥ 85^th^ percentile and < 95^th^ percentile; healthy weight ≥ 5^th^ percentile and < 85^th^ percentile; and underweight < 5^th^ percentile. Region of residence was separated into 3 groups by administrative district: large cities, small cities, and rural areas. Income was grouped into 5 levels ranging from highest to lowest. Parental education level was classified into 4 groups: graduated from college or above; graduated from high school; graduated from middle school or under; and unknown or no parent. Participants who did not know the educational level of their parents or who did not have a parent were not excluded, as their exclusion could increase the number of missing values among participants with a lower income level. The self-reported stress level of participants was categorized into 5 groups: severe, moderate, mild, a little, and no stress. The participants were asked about the number of days that they had smoked in the past one month, and this value was separated into 4 groups: 0 days a month; 1–5 days a month; 6–19 days a month; and ≥ 20 days a month. The participants were also asked to provide the number of days that they had consumed alcohol in the past month, which was then categorized into 3 groups: 0 days a month; 1–5 days a month; and 6–30 days a month. Finally, participants were asked about their history of drug or substance use. Total study time was classified into 4 groups: ≤ 2 hours a day; > 2 hours and ≤ 6 hours a day; > 6 hours and ≤ 9 hours a day; and > 9 hours a day.

#### Independent variables: Classification of internet use

Internet use for study was measured in hours and 10-minute increments. Mean daily internet use was calculated by adding the time spent on weekdays and the time spent on weekends, using a 5/7 weight and 2/7 weight, respectively. We separated internet use for study into 4 groups (0 hours a day [0 h]; > 0 and ≤ 1 hour a day [1 h]; > 1 and ≤ 2 hours a day [2 h]; and > 2 hours a day [2+ h]) and internet use for general purpose into 5 groups (0 h; 1 h; 2 h; > 2 and ≤ 3 hours a day [3 h]; and > 3 hours a day [3+ h]), including the 3+ h group, because only a few of the participants had used the internet for study for more than 3 hours a day.

#### Dependent variables: Classification of school performance

The participants were asked about their study performance at school in the last 12 months. Self-reported school performance was classified into 5 groups: A (highest); B (middle, high); C (middle); D (middle, low); and E (lowest).

### Statistical analysis

The differences in general characteristics according to school performance were calculated using linear regression analysis with complex sampling and the Chi-square test with the Rao-Scott correction. Odds ratios (ORs) of internet use for study/ general purpose with respect to school performance were calculated using simple logistic regression analysis with complex sampling (unadjusted); multinomial logistic regression analysis with complex sampling adjusted for age, sex, obesity, region of residence, income level, education level of father, education level of mother, stress level, sleep time, days of physical activity, smoking, alcohol consumption, drug use, and total study time (model 1); and multinomial logistic regression analysis with complex sampling adjusted for the model 1 variables as well as internet use for study or other purposes (model 2).

For the subgroup analysis according to income level, adjusted ORs (AORs) of internet use for study/other purposes with respect to school performance were calculated via multinomial logistic regression analysis with complex sampling using model 2.

Two-tailed analyses were conducted, and *P*-values lower than 0.05 were considered to indicate significance. Additionally, 95% confidence intervals (CIs) were calculated. All results are presented as weighted values after applying the weightings recommended by the KYRBWS. The results were analysed using SPSS ver. 21.0 (IBM, Armonk, NY, USA).

## Results

In total, 11.1%, 24.4%, 28.1%, 24.7%, and 11.8% of the participants were grouped into performance group A, B, C, D, and E, respectively (Table 1). All of the considered variables including age, days of physical activity, sex, obesity, region of residence, level of income, level of parental education, stress, sleep time, smoking, alcohol consumption, drug use, and total study time significantly differed between the performance groups (P < 0.001 for each variable). Internet use times for study and general purpose showed significant differences between the school performance groups (each P < 0.001).

**Table 1:** General characteristics of participants according to performance at school.

Factors	Total	Performance at School					P-value
		A	B	C	D	E	
Number							
n	59,105	6,550	14,410	16,6117	14,581	6,947	
%	100	11.1	24.4	28.1	24.7	11.8	
Mean Age (year)	15.0	14.7	14.9	15.1	15.1	15.1	<0.001*
Physical Activity (day)	2.87	3.05	2.93	2.81	2.81	2.81	<0.001*
Sex (%)							<0.001†
Male	51.7	57.7	50.3	50.3	50.4	55.1	
Female	48.3	42.3	49.7	49.7	49.6	44.9	
Obesity (%)							<0.001†
Underweight	6.0	6.0	5.5	5.9	6.5	6.7	
Healthy	79.4	80.4	80.8	80.4	77.7	76.7	
Overweight	11.2	10.8	10.7	10.5	12.2	12.1	
Obese	3.4	2.8	3.0	3.3	3.6	4.7	
Region (%)							<0.001†
Large City	44.2	47.4	46.2	43.7	43.0	40.9	
Small City	49.1	45.8	47.4	49.5	50.4	52.0	
Rural Area	6.7	6.9	6.4	6.8	6.6	7.1	
Income Level (%)							<0.001†
Highest	7.0	24.5	7.4	3.7	3.9	4.4	
Middle High	25.1	32.8	37.2	23.7	16.9	13.1	
Middle	48.0	31.7	41.5	56.2	53.3	46.2	
Middle Low	15.8	8.8	11.9	14.2	21.5	23.1	
Lowest	4.0	2.3	2.1	2.2	4.5	13.3	
Education, Father (%)							<0.001†
Unknown	18.1	9.8	12.1	17.2	23.1	30.6	
Middle School	3.3	2.0	2.7	2.9	3.8	5.4	
High School	32.1	22.5	29.3	34.2	35.5	35.2	
College, or over	46.5	65.7	55.9	45.7	37.6	28.8	
Education, Mother (%)							<0.001†
Unknown	17.2	9.3	11.7	15.7	22.0	29.6	
Middle School	3.1	1.9	2.6	3.0	3.7	4.5	
High School	41.8	32.9	39.9	44.1	44.8	42.5	
College, or over	37.9	55.9	45.8	37.1	29.5	23.4	
Stress (%)							<0.001†
Severe	10.9	9.1	8.9	9.3	12.0	18.7	
Moderate	30.3	26.1	28.8	29.9	32.5	33.4	
Mild	41.8	41.3	43.9	44.3	40.6	34.2	
A little	14.2	18.7	15.9	14.0	12.5	10.7	
No	2.8	4.9	2.5	2.5	2.4	3.0	
Sleep time (%)							<0.001†
< 5.5 h	24.9	23.9	24.8	25.6	23.9	26.2	
≥ 5.5 h, <6.5 h	24.7	26.0	25.1	28.4	24.2	23.1	
≥ 6.5 h, <7.5 h	24.8	24.3	25.2	24.6	24.8	25.1	
≥ 7.5 h	25.7	25.8	24.8	25.0	27.1	25.7	
Smoking (%)							<0.001†
No	90.8	95.5	94.7	92.3	88.8	78.5	
1–5 days/month	2.3	1.4	1.4	1.9	3.0	4.8	
6–19 days/month	1.4	0.9	1.0	1.3	1.8	2.6	
20–30 days/month	5.4	2.3	3.0	4.4	6.4	14.1	
Alcohol (%)							<0.001†
No	84.0	88.4	86.8	85.3	82.4	74.5	
1–5 days/month	12.3	9.4	10.8	11.7	13.5	17.6	
6–30 days/month	3.6	2.2	2.4	3.1	4.2	7.9	
Drugs (%)							<0.001†
No	99.3	99.2	99.5	99.4	99.3	99.0	
Yes	0.7	0.8	0.5	0.6	0.7	1.0	
Total Study time							<0.001†
≤ 2 h	23.3	13.8	16.4	22.0	28.6	38.9	
> 2 h, ≤ 6h	23.9	20.8	22.7	23.7	25.6	26.7	
> 6 h, ≤ 9 h	24.9	26.1	27.1	25.0	24.0	20.7	
> 9 h	27.9	39.3	33.8	29.3	21.8	13.7	
Internet for study							<0.001†
0 h	50.0	38.6	42.4	48.4	55.8	69.0	
1 h	28.9	33.6	32.9	30.3	26.1	18.0	
2 h	12.5	16.2	14.6	12.8	10.9	7.1	
2+ h	8.6	11.6	10.1	8.5	7.1	5.9	
Internet for general purpose							<0.001†
0 h	25.3	23.8	22.6	24.8	26.1	31.5	
1 h	28.1	35.6	31.7	28.9	24.4	19.6	
2 h	21.1	21.6	23.1	21.9	20.2	16.7	
3 h	13.0	10.5	12.4	12.9	14.5	13.4	
3+ h	12.5	8.4	10.2	11.5	14.8	18.8	

*Linear regression analysis with complex sampling, Significance at P < 0.05

^†^ Chi-square test with Rao-Scott correction, Significance at P < 0.05

Compared to the lowest school performance group (E), internet use for study of 2+ h was related to higher school performance in the unadjusted model, model 1, and model 2 (AOR [95% CI] for internet use for study of 2+ h in model 2 = 2.43 [2.10–2.82], 2.02 [1.78–2.30], 1.66 [1.46–1.89], and 1.30 [1.15–1.47] for performance groups A, B, C, and D, respectively, P < 0.001). Additionally, 2 h of internet use for study was associated with higher school performance, with a slightly higher AOR (for internet use 2 h for study of 2h = 2.71 [2.40–3.07], 2.27 [2.04–2.53], 1.92 [1.73–2.14], and 1.55 [1.39–1.72] for performance groups A, B, C, and D, respectively, P < 0.001). Moreover, internet use for study of only 1 h was associated with higher school performance (AOR for internet use for study of 1 h = 2.38 [2.16–2.62], 2.12 [1.95–2.30], 1.85 [1.70–2.00], and 1.49 [1.38–1.61] for performance groups A, B, C, and D, respectively, P < 0.001) (Table 2).

**Table 2:** Odd ratios of internet using time for school performance (Reference: 0 h use of internet, the lowest school performance, E).

Model	**Internet Use for Study**					
	Unadjusted		Model 1		Model 2	
	OR (95% CI)	P-value	AOR (95% CI)	P-value	AOR (95% CI)	P-value
Performance, A		<0.001*		<0.001*		<0.001*
2+ h	3.54 (3.09–4.06)		2.41 (2.09–2.78)		2.43 (2.10–2.82)	
2 h	4.09 (3.65–4.58)		2.80 (2.48–3.15)		2.71 (2.40–3.07)	
1 h	3.32 (3.04–3.63)		2.53 (2.31–2.77)		2.38 (2.16–2.62)	
0 h	1		1		1	
Performance, B						
2+ h	2.82 (2.50–3.18)		2.07 (1.82–2.34)		2.02 (1.78–2.30)	
2 h	3.35 (3.03–3.71)		2.39 (2.15–2.66)		2.27 (2.04–2.53)	
1 h	2.97 (2.75–3.21)		2.27 (2.09–2.46)		2.12 (1.95–2.30)	
0 h	1		1		1	
Performance, C						
2+ h	2.07 (1.84–2.34)		1.67 (1.48–1.89)		1.66 (1.46–1.89)	
2 h	2.57 (2.32–2.84)		1.98 (1.79–2.20)		1.92 (1.73–2.14)	
1 h	2.40 (2.22–2.59)		1.93 (1.78–2.09)		1.85 (1.70–2.00)	
0 h	1		1		1	
Performance, D						
2+ h	1.51 (1.34–1.70)		1.32 (1.17–1.50)		1.30 (1.15–1.47)	
2 h	1.90 (1.71–2.10)		1.60 (1.44–1.78)		1.55 (1.39–1.72)	
1 h	1.79 (1.66–1.93)		1.54 (1.43–1.67)		1.49 (1.38–1.61)	
0 h	1		1		1	
Model	**Internet Use for general purpose**					
	Unadjusted		Model 1		Model 2	
	OR (95% CI)	P-value	AOR (95% CI)	P-value	AOR (95% CI)	P-value
Performance, A		<0.001*		<0.001*		<0.001*
3+ h	0.59 (0.52–0.67)		0.86 (0.75–0.98)		0.68 (0.60–0.78)	
3 h	1.03 (0.91–1.18)		1.12 (0.98–1.29)		0.85 (0.74–0.98)	
2 h	1.72 (1.54–1.91)		1.54 (1.37–1.72)		1.13 (1.00–1.28)	
1 h	2.40 (2.17–2.65)		1.90 (1.71–2.11)		1.42 (1.27–1.59)	
0 h	1		1		1	
Performance, B						
3+ h	0.75 (0.69–0.83)		1.02 (0.92–1.14)		0.85 (0.76–0.94)	
3 h	1.29 (1.17–1.43)		1.33 (1.20–1.47)		1.05 (0.95–1.17)	
2 h	1.93 (1.75–2.13)		1.67 (1.51-.185)		1.30 (1.17–1.44)	
1 h	2.25 (2.07–2.45)		1.74 (1.59–1.90)		1.37 (1.25–1.49)	
0 h	1		1		1	
Performance, C						
3+ h	0.78 (0.71–0.85)		0.96 (0.87–1.05)		0.83 (0.75–0.92)	
3 h	1.22 (1.11–1.34)		1.20 (1.08–1.32)		1.00 (0.90–1.11)	
2 h	1.67 (1.54–1.82)		1.44 (1.32–1.57)		1.18 (1.08–1.29)	
1 h	1.87 (1.73–2.03)		1.49 (1.47–1.62)		1.23 (1.13–1.34)	
0 h	1		1		1	
Performance, D						
3+ h	0.96 (0.87–1.04)		1.07 (0.97–1.18)		0.98 (0.89–1.08)	
3 h	1.31 (1.20–1.44)		1.28 (1.17–1.41)		1.15 (1.05–1.27)	
2 h	1.46 (1.34–1.60)		1.33 (1.21–1.45)		1.17 (1.07–1.29)	
1 h	1.50 (1.38–1.64)		1.30 (1.19–1.41)		1.15 (1.06–1.26)	
0 h	1		1		1	

* Statistical significance < 0.05

Internet use for general purpose showed a negative relationship with school performance. Using the internet for general purpose for 3+ h was negatively related to higher school performance in the unadjusted model, model 1, and model 2 (AOR for internet use for general purpose of 3+ h in model 2 = 0.68 [0.60–0.78], 0.85 [0.76–0.94], 0.83 [0.75–0.92], and 0.98 [0.89–1.08] for performance groups A, B, C, and D, respectively, P < 0.001). Furthermore, 3 h of internet use for general purpose was negatively associated with higher school performance (AOR for internet use for general purpose of 3 h = 0.85 [0.74–0.98], 1.05 [0.95–1.17], 1.00 [0.90–1.11], and 1.15 [1.05–1.27] for performance groups A, B, C, and D, respectively, P < 0.001). However, relatively short use of the internet for general purpose (2 h or 1 h a day) was positively associated with school performance (AOR for internet use for general purpose of 2 h = 1.13 [1.00–1.28], 1.30 [1.17–1.44], 1.18 [1.08–1.29], and 1.17 [1.07–1.29]; AOR for internet use for general purpose of 1 h = 1.42 [1.27–1.59], 1.37 [1.25–1,49], 1.23 [1.13–1.34], and 1.15 [1.06–1.26] for performance groups A, B, C, and D, respectively, each P < 0.001). In the subgroup analysis according to income level, the associations between internet use and school performance were consistent; this relationship was strongest and most evident in the lowest income group, and it was less clear in the highest income group (Table 3). Using the internet for study for 2+ h showed high AORs in the lowest income group (3.23 [1.83–5.71], 1.40 [0.87–2.26], 1.49 [1.01–2.21], and 1.31 [0.88–1.94] for performance groups A, B, C, and D, respectively, P < 0.001) but showed lower AORs in the highest income group (1.49 [0.94–2.35], 1.29 [0.78–2.11], 0.96 [0.55–1.67], and 1.29 [0.73–2.28] for performance groups A, B, C, and D, respectively).

**Table 3:** Subgroup analysis of adjusted odd ratios of internet using time for school performance (Reference: 0 h use of internet, the lowest school performance, E) according to income level.

Income	**Internet Use for Study**									
	Highest		Upper-middle		Middle		Low-middle		Lowest	
	AOR (95% CI)	P-value	AOR (95% CI)	P-value	AOR (95% CI)	P-value	AOR (95% CI)	P-value	AOR (95% CI)	P-value
Performance, A		0.002*		<0.001*		<0.001*		<0.001*		<0.001*
2+ h	1.49 (0.94–2.35)		2.88 (2.05–4.03)		2.62 (2.07–3.30)		2.24 (1.50–3.33)		3.23 (1.83–5.71)	
2 h	2.14 (1.33–3.43)		3.89 (2.82–5.38)		2.76 (2.30–3.31)		2.93 (2.14–4.02)		2.95 (1.75–4.96)	
1 h	1.86 (1.33–2.60)		2.35 (1.89–2.91)		2.57 (2.23–2.95)		2.58 (2.03–3.27)		2.49 (1.58–3.94)	
0 h	1		1		1		1		1	
Performance, B										
2+ h	1.29 (0.78–2.11)		2.21 (1.61–3.04)		2.32 (1.89–2.85)		1.60 (1.19–2.15)		1.40 (0.87–2.26)	
2 h	2.25 (1.39–3.64)		2.90 (2.14–3.93)		2.31 (1.98–2.71)		2.12 (1.61–2.79)		1.55 (0.96–2.51)	
1 h	1.94 (1.36–2.76)		2.00 (1.65–2.42)		2.19 (1.94–2.47)		2.12 (1.77–2.56)		1.92 (1.37–2.71)	
0 h	1		1		1		1		1	
Performance, C										
2+ h	0.96 (0.55–1.67)		1.85 (1.34–2.57)		1.74 (1.42–2.13)		1.62 (1.26–2.08)		1.49 (1.01–2.21)	
2 h	2.12 (1.31–3.43)		2.61 (1.91–3.57)		1.84 (1.58–2.14)		1.93 (1.52–2.46)		1.53 (0.98–2.40)	
1 h	1.78 (1.20–2.64)		1.70 (1.39–2.07)		1.93 (1.72–2.16)		1.78 (1.50–2.10)		1.97 (1.28–2.73)	
0 h	1		1		1		1		1	
Performance, D										
2+ h	1.29 (0.73–2.28)		1.27 (0.91–1.79)		1.36 (1.13–1.65)		1.26 (0.99–1.61)		1.31 (0.88–1.94)	
2 h	1.50 (0.88–2.58)		1.89 (1.37–2.61)		1.60 (1.37–1.87)		1.41 (1.11–1.79)		1.39 (0.93–2.07)	
1 h	1.62 (1.10–2.40)		1.38 (1.13–1.70)		1.45 (1.29–1.62)		1.64 (1.39–1.93)		1.74 (1.31–2.33)	
0 h	1		1		1		1		1	
Income	**Internet Use for general purpose**									
	Highest		Upper-middle		Middle		Low-middle		Lowest	
	AOR (95% CI)	P-value	AOR (95% CI)	P-value	AOR (95% CI)	P-value	AOR (95% CI)	P-value	AOR (95% CI)	P-value
Performance, A		<0.001*		<0.001*		<0.001*		<0.001*		0.217
3+ h	0.48 (0.30–0.78)		0.60 (0.44–0.82)		0.70 (0.56–0.88)		0.91 (0.65–1.29)		0.74 (0.41–1.33)	
3 h	0.34 (0.22–0.53)		0.89 (0.65–1.22)		0.98 (0.80–1.20)		1.26 (0.91–1.76)		0.73 (0.37–1.43)	
2 h	0.51 (0.32–0.81)		1.38 (1.06–1.80)		1.22 (1.02–1.46)		1.41 (1.06–1.88)		1.02 (0.57–1.84)	
1 h	0.62 (0.43–0.91)		1.83 (1.43–2.35)		1.46 (1.24–1.73)		2.06 (1.57–2.72)		1.38 (0.83–2.30)	
0 h	1		1		1		1		1	
Performance, B										
3+ h	0.65 (0.39–1.06)		0.65 (0.50–0.84)		0.84 (0.72–0.98)		1.13 (0.90–1.41)		1.17 (0.77–1.78)	
3 h	0.62 (0.39–0.97)		0.93 (0.71–1.21)		1.08 (0.92–1.26)		1.48 (1.18–1.87)		1.06 (0.63–1.77)	
2 h	0.90 (0.56–1.45)		1.17 (0.92–1.48)		1.34 (1.16–1.54)		1.60 (1.27–2.01)		1.17 (0.75–1.82)	
1 h	0.94 (0.63–1.39)		1.43 (1.14–1.80)		1.34 (1.18–1.52)		1.56 (1.26–1.94)		1.33 (0.92–1.92)	
0 h	1		1		1		1		1	
Performance, C										
3+ h	0.75 (0.44–1.26)		0.70 (0.55–0.90)		0.75 (0.65–0.87)		1.02 (0.84–1.25)		1.36 (0.93–2.00)	
3 h	0.68 (0.41–1.13)		1.05 (0.80–1.37)		0.92 (0.79–1.06)		1.33 (1.07–1.66)		1.06 (0.69–1.64)	
2 h	0.80 (0.48–1.33)		1.19 (0.95–1.49)		1.09 (0.96-.124)		1.51 (1.25–1.82)		1.30 (0.86–1.97)	
1 h	0.75 (0.48–1.16)		1.38 (1.09–1.75)		1.14 (1.01–1.28)		1.49 (1.24–1.79)		1.33 (0.90–1.96)	
0 h	1		1		1		1		1	
Performance, D										
3+ h	0.76 (0.45–1.26)		1.05 (0.82–1.35)		0.92 (0.80–1.06)		0.97 (0.80–1.16)		1.46 (1.06–2.01)	
3 h	0.74 (0.46–1.20)		1.20 (0.93–1.56)		1.09 (0.94–1.26)		1.36 (1.12–1.66)		1.36 (0.95–1.94)	
2 h	0.97 (0.60–1.55)		1.31 (1.03–1.67)		1.08 (0.95–1.23)		1.22 (1.01–1.47)		1.58 (1.14–2.18)	
1 h	0.64 (0.41–1.01)		1.36 (1.07–1.74)		1.08 (0.95–1.22)		1.26 (1.06–1.50)		1.44 (1.05–1.99)	
0 h	1		1		1		1		1	

* Statistical significance < 0.05

## Discussion

Internet use for study showed significant positive correlations with higher school performance, and these positive relations were highest in students who spent 2 hours a day using the internet for academic purposes. The subject with 2 hours a day of internet use for study demonstrated 2.71 times more group A school performance, compared to none internet user for study. In contrast, internet use for general purpose was associated with lower school performance. The subject with 3 or more hours a day of internet use for general purpose showed 0.68 times less group A school performance, compared to none internet user for general purpose. The beneficial correlation of internet use for study with school performance and the detrimental correlation of internet use for general purpose with school performance remained significant even after adjusting for other variables including age, physical activity, sex, obesity, region of residence, income level, parental education level, stress level, sleep time, smoking, alcohol consumption, drug use, and total study time. In the subgroup analyses by income level, the positive associations between school performance and internet use for study were weakest in the highest income group and were strongest in the lowest income group. Regardless of income level, excessive use of the internet for general purpose was related to lower school performance. The present study extended previous studies that found negative or controversial associations between academic performance and internet use by identifying a positive correlation between internet use for study and school performance based on quantitatively categorized data. The primary strength of this study is that it was based on data from a very large population. Additionally, we considered numerous factors that could be related to school performance to minimize potential confounding effects. Most importantly, these results have merits to encompass a wide range of internet use durations, from 0 hours to more than 3 hours, and to investigate the effects of internet use time on school performance, which was also classified into 5 levels. The sampling design and adjusted weighting increased the statistical power of our results.

### Plausible reasons for favourable association of internet use for study with school performance

Internet use for study showed a positive association with school performance in this study. Recent studies demonstrated the beneficial effects of social media use for study on school performance [28], while other studies did not show significant correlation between educational use of internet and school performance [26,27]. By including every sort of internet contents for educational purpose, not restricted to social media networking, the present study revealed the discriminated, positive relation between internet use for study and school performance. Although several previous studies suggested a negative association between higher school performance and overuse or problematic internet use, they did not incorporate the reason for the internet use or considered only cases of overuse of more than 3 hours a day [9,14]. In accordance with these previous reports, our results also demonstrated that 2 h of internet use for academic purposes had a higher OR than 2 h+ of internet use for academic purposes. We believe that using the internet for excessive periods, even for studying, could impede school performance. However, there are several studies that advocate for learning using online networks [34–36]. Multiple plausible hypotheses have been provided for this approach, including connectivism, which presumes that learning phenomena involve a network of technology and socialization [34]. In this theory, the attainment of knowledge is explained by a process of localization of information, acquisition of content, development and recollection of a recurrent pattern in the networks [37]. The vast renewal of information and the interactive content available on the internet have the potential to promote all of these learning process. Because diverse information and opinions are available, it may be important to learn how to selectively connect these data [38]. Internet networking can facilitate the navigation and filtering of these available data. Through online communication, students can participate and collaborate in their own learning process as well as receive feedback from instructors [35,36]. Furthermore, the completion of online academic courses and the use of collective student blogs may foster individuals’ autonomy in learning [34]. In fact, digital learning has been increasingly applied to diverse medical education systems, showing beneficial effects [39,40].

### Adverse aspects of internet use for general purpose on school performance

In contrast, internet use for general purpose had a negative correlation with school performance in our study, even after considering other possible confounding factors. The negative association between internet use for general purpose and school performance can be explained by several mental and physical changes that occur due to internet use. Intensive use of the internet leads to behavioural problems that demonstrate an aggressive, compulsive and addictive pattern [41–43]. Many internet media including blogs and games can induce compulsiveness by continually requiring users to participate and update their information in order to avoid falling behind. Thus, if appropriately used, interned media can be helpful for sustaining attention, memory and social communication skills. However, when excessively used, they may interfere with school work and sleep time and, if chronically continued, may result in an imbalance of circadian rhythms [23]. Furthermore, internet users are continuously exposed to new messages or data, and this constant exposure can interrupt their concentration on schoolwork. Multi-tasking is often required during internet networking due to the intrusiveness of social message services, pop-up advertisements, and linked information pages. Finally, previous studies have demonstrated the occurrence of subsequent mental problems including depressive symptoms, loneliness, anxiety, and low self-esteem after excessive internet use in adolescents [44–46].

### Positive aspects of internet use for general purpose on school performance

However, internet use for general purpose is not always related to poor school performance. Compared to non-use, short (1 h) use of the internet for general purpose has demonstrated positive associations with higher school performance. Several beneficial aspects of internet use could explain this positive relationship between higher school performance and internet use. Using the internet for entertainment or social networking may re-energize a student from the burden and stress imposed by schoolwork. It is well known that stress can have detrimental effects on spatial learning and memory and that an enriched environment can reverse these adverse effects [47]. Furthermore, it is possible that using the internet might be a more favourable source of socioeconomic support for achieving better school performance than not using the internet. Although we accounted for socioeconomic factors such as income level, parental education level, and region of residence, it is plausible that other confounders of socioeconomic factors were not adjusted for in the analyses.

Our results demonstrated that the effects and purposes of internet use differed according to income level. A previous study suggested that income influenced internet use and academic performance individually but did not affect the association between using the internet and academic performance [40]. However, their results were limited to children with low grades, indicating that greater internet use was related to better reading skills and visual-spatial skills only in youth with poor reading skills and a low grade point average [40]. In consistent to our results, prior study showed the improvement of math test scores by using computer learning programs in low income groups [48]. In this study, the highest income group showed an attenuated correlation between higher school performance and internet use for academic purposes. Although poor income groups may not use the internet as readily, as mentioned above, the internet is widely used due to its relatively low cost and easy accessibility compared to expensive private lessons, especially in Korea. A longitudinal study of 140 children demonstrated that greater internet use was related to higher academic performance in individuals with an annual income of 15,000 (U.S. dollars) or less [41,42]. Because individuals in the highest income groups have abundant educational resources, they may not receive additional benefits from learning through the internet. However, other income groups can more easily access the relatively cheap and qualified education available through the internet. For instance, various online educational videos can be easily accessed free of charge at any time, suggesting that learning through the internet could be a means of overcoming the rich-poor gap in education and could be particularly helpful to low income groups.

### Limitations of the present study

However, the present study used a cross-sectional design, and therefore, causality could not be established. The use of anonymous self-reporting limited the potential for selection and reporting biases. School performance and time using the internet were obtained by self-report, which might limit the accuracy of the data. We used time of internet use as a parameter for the amount of internet use, without considering dependence, compulsiveness or other mental or physical problems that may be related to internet use. However, by quantifying adolescents’ internet use and categorizing the time spent in detail, we were able to investigate the possible effects of a wide range of internet use, from 0 to more than 2 hours a day, on academic performance, thus including cases other than the extreme cases of internet addiction that had previously been analysed. Moreover, previous studies support our findings that longer internet use closely correlated with adverse effects of internet use [14]. Further studies with a prospective study design that consider additional factors potentially related to internet use or school performance will reveal the causality between internet use and school performance.

## Conclusion

Higher school performance was significantly associated with internet use for study, with the strongest association for 2 hours of internet use for study per day. In contrast, internet use for general purpose showed a negative correlation with higher school performance. The positive association between higher school performance and internet use for study was weakest in the highest income group and strongest in the lowest income groups. The relatively low cost of the internet as a medium for providing education in Korea may explain the differential associations between internet use and school performance by income group. Therefore, internet use for academic purposes is presumed to be a versatile tool that could reduce the economic inequalities in education.
